# Study on the Consistency of Elements in 1-1-3-Type Piezoelectric Composite Materials

**DOI:** 10.3390/s25051479

**Published:** 2025-02-27

**Authors:** Zhongrui Du, Nianyi Shen, Chao Zhong, Lei Qin

**Affiliations:** Beijing Key Laboratory for Sensor, Beijing Information Science & Technology University, Beijing 100192, China; 2022020326@bistu.edu.cn (Z.D.); 18164043192@163.com (N.S.); 20192289@bistu.edu.cn (C.Z.)

**Keywords:** piezoelectric array, element consistency, vibration modes, electromechanical coupling coefficient

## Abstract

This study introduces a novel 1-1-3-type piezoelectric array structure and investigates variations in the piezoelectric phase’s volume fraction. Its performance and consistency are compared with that of a conventional 1-3-type piezoelectric array of identical volume fraction. Finite element analysis was applied to study the effects of the positions of the elements in 1-1-3-type and 1-3-type piezoelectric arrays on the electrical conductivity curves, as well as the differences in vibration modes. To validate the theoretical models, experimental fabrication and testing were performed, and we developed a high-precision testing fixture designed to minimize experimental errors. The results demonstrate that the resonance frequency fluctuations in the 1-1-3-type piezoelectric array are maintained within 1%, and conductance fluctuations within 13.4%, significantly enhancing consistency compared to the 1-3-type array. Furthermore, the electromechanical coupling coefficient of the 1-1-3 array was also found to be superior to that of the 1-3-type, indicating improved performance parameters.

## 1. Introduction

High-frequency (greater than 100 KHz) imaging sonar, as a crucial piece of underwater acoustic detection equipment, utilizes high-frequency sound waves to generate images or three-dimensional models of underwater environments. It is extensively used in applications such as underwater salvage, mine hunting, and seabed topography exploration [1,2]. As oceanic exploitation has intensified in recent years, the demand for high-frequency, high-precision imaging sonar has grown significantly. On one hand, this requires algorithms to be more accurate and efficient; on the other hand, it demands higher performance from transducer hardware. Consistency indicates the convergence of the performance of each element in the array, which is the basic requirement of the piezoelectric transducer array in the high-frequency sonar system. The consistency of the transducer array significantly impacts the sonar’s performance and is essential for improving imaging accuracy [3].

Currently, high-frequency imaging sonar transducer arrays generally use 1-3-type piezoelectric composites. On one hand, high-frequency imaging sonar demands higher performance from piezoelectric composites in terms of the electromechanical coupling coefficient, acoustic impedance, the hydrostatic piezoelectric constant, and other properties, and researchers have made significant progress in this area [4,5,6,7,8,9,10,11,12,13,14,15,16,17,18,19,20,21,22]. Liu Shengwen et al. from Central South University prepared a 1-3-type PZT/epoxy resin composite, which exhibited a thickness electromechanical coupling coefficient of about 0.61, with a variation of less than 1% within the temperature range of −20 to 60 °C, demonstrating good temperature stability [23]. Li Feng from Hefei University of Technology prepared a lead-free piezoelectric ceramic/epoxy resin 1-3-type composite based on sodium bismuth titanate, which exhibited an electromechanical coupling coefficient of 0.547 and a relative dielectric constant of 128.4 at a ceramic volume fraction of 27.6%, with an acoustic impedance of 9 Mrayls [24]. Wang Wei and colleagues at Xi’an Jiaotong University used a 0.2PIN-0.35PMN-0.33PT (PIMNT) system to fabricate a 1-3-type composite with a ceramic volume fraction of 60%, achieving an electromechanical coupling coefficient of 0.84, an acoustic impedance of 19 Mrayls, and a piezoelectric constant of 1256 C/N [25]. Li Ning and others proposed a paradigm for modulating vibration modes by adjusting the polymer ratio, obtaining an electromechanical coupling coefficient of 0.69 when the ceramic volume fraction of (PZT4) was 24.5% [26]. Mi et al. prepared 1-1-3-type piezoelectric composites using piezoelectric ceramics, epoxy resin, and silicone rubber, achieving an electromechanical coupling coefficient of 0.69, while reducing the acoustic impedance to 6.81 Mrayls, making it a suitable material for high-frequency underwater sonar transducers [27].

On the other hand, high-frequency imaging sonar transducers require higher consistency among the elements. The array-casting method is an early technique used for fabricating piezoelectric composites. Liu Dianfeng et al., from the Institute of Acoustics of the Chinese Academy of Sciences, used this method to produce 1-3-type piezoelectric composites. They explored the non-uniform excitation modes of transducers and conducted theoretical analyses and experimental designs on edge Gaussian and Fresnel focusing transducers, concluding that the piezoelectric phase volume ratio can alter the transducer’s radiation degree and that changes in the polarization direction of the piezoelectric phase can affect the phase of the radiating surface [28]. Typically, this method uses a positioning device to determine the locations, then selecting base elements with good consistency to form the array. However, for piezoelectric arrays requiring frequencies above 500 kHz, where element spacing is further reduced, the demands for precision are extremely high, making the array-casting method less suitable. For such cases, the cut-injection technique is commonly used, which involves forming a 1-3-type piezoelectric array by segmenting electrodes [29]. Based on this technique, Chen Jing proposed an “integrated molding” fabrication process for producing 1-1-3-type piezoelectric arrays, significantly enhancing fabrication precision and improving element consistency [30].

This paper fabricated piezoelectric arrays using the previously studied 1-1-3-type piezoelectric composites, investigating their element consistency through simulation and physical production, and also designed a test fixture that can reduce testing errors. To compare the performance advantages of the 1-1-3-type piezoelectric array, a 1-3-type piezoelectric array was simultaneously produced, with the piezoelectric pillar size, number, and spacing identical to those of the 1-1-3 array. This study concludes that the 1-1-3-type piezoelectric transducers exhibit better overall element consistency.

## 2. Structure of the 1-1-3-Type Piezoelectric Composite Material

**Figure 1:**
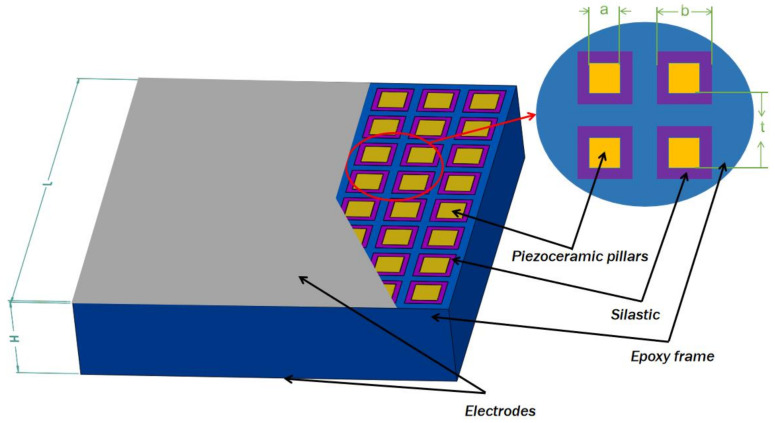
Structure of the 1-1-3-type piezoelectric composite material.

The structure of the 1-1-3-type (the first 1 represents that the piezoelectric phase has connectivity in one direction, and the second 1 and 3 represent that the two polymer phases have connectivity in one and three directions, respectively) piezoelectric composite material designed in this study is shown as Figure 1:

The array is composed of piezoelectric ceramics, silicone rubber, epoxy resin, and silver electrodes. The overall thickness H of the array is 7.5 mm, and both the length and width L are 34.4 mm each. It includes an 8 × 8 grid of elements, with a fixed inter-element spacing t of 4.2 mm. The square holes within the epoxy resin framework have a fixed side length b of 3.4 mm. The cross-sectional side length a of each piezoelectric ceramic pillar can vary among 2.0 mm, 2.2 mm, and 2.4 mm, with the arrays referred to as number 1, 2, and 3, respectively. Silicone rubber is used to fill the gaps between the piezoelectric ceramic pillars and the epoxy resin frame.

**Figure 2:**
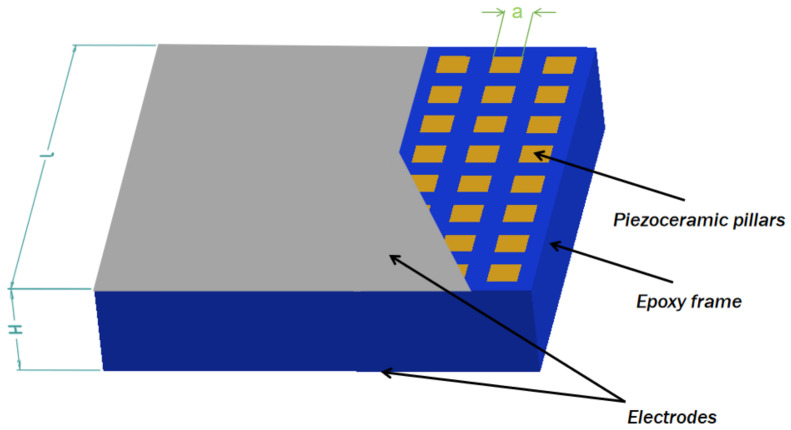
Structure of the 1-3-type piezoelectric composite material.

To compare the performance of the 1-1-3-type piezoelectric composite material, this paper also simultaneously prepares a 1-3-type (1 indicates that the piezoelectric phase has connectivity in one direction, and 3 indicates that the polymer phase has connectivity in three directions) piezoelectric composite material, whose structure is shown as Figure 2.

Consistency with the aforementioned 1-1-3-type piezoelectric composite material is ensured for the overall array dimensions, inter-element spacing, and number of elements. Additionally, the cross-sectional side length a of each piezoelectric ceramic pillar can also vary among 2.0 mm, 2.2 mm, and 2.4 mm, with these arrays referred to as number 4, 5, and 6, respectively.

## 3. Finite Element Simulation of Piezoelectric Composite Materials

Finite element analysis of both the 1-1-3- and 1-3-type piezoelectric composite materials was conducted using ANSYS software (2323 R2). The piezoelectric phase material selected was PZT-5A, with its material parameters listed in Table 1 [31]. The polymer phase materials selected were Nanda® 704 (Liyang, China) silicone rubber and E-51 epoxy resin. The performance parameters for silicone rubber and epoxy resin are provided in Table 2.

**Figure 3:**
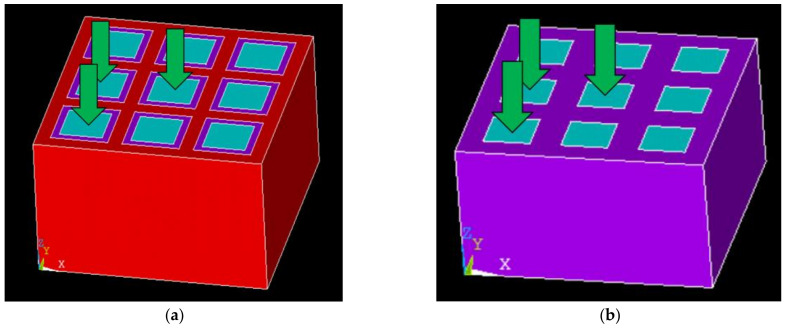
Finite element simulation models of piezoelectric composite materials. (**a**) Simulation model of 1-1-3-type piezoelectric composite material and selection of piezoelectric pillars; (**b**) simulation model of 1-3-type piezoelectric composite material and selection of piezoelectric pillars.

**Figure 4:**
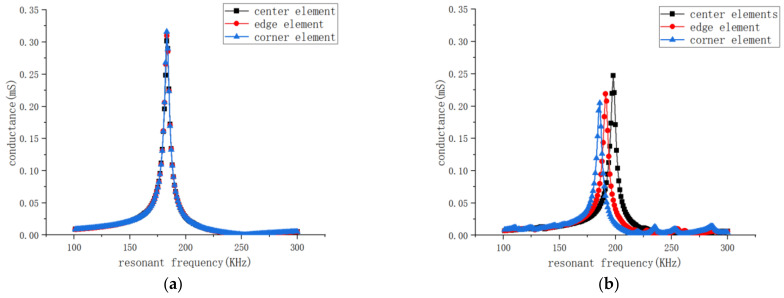
Simulation results of admittance curves. (**a**) Admittance curve simulation results for edge, corner, and center elements in the 1-1-3-type array. (**b**) Admittance curve simulation results for edge, corner, and center elements in the 1-3-type array.

Considering the grid refinement and the model size requirements on computer performance, only a 3 × 3 array is simulated in this study to illustrate the issue. The simulated array has a thickness of 7.5 mm, with cross-sectional dimensions of the piezoelectric pillars of 2.4 mm × 2.4 mm and a step size of 4 mm. The cell type of the piezoelectric phase element is solid 5, which has eight nodes, each with up to 6 degrees of freedom. And the cell type of the polymer phase is solid 185, which has eight nodes, each with 3 degrees of freedom parallel to the XYZ axis. As shown in Figure 3, piezoelectric pillars are selected from three positions (edge, corner, and center) in both the 1-1-3 and 1-3 piezoelectric composite materials. A harmonic voltage excitation of 1V and 0V is applied to the upper and lower surfaces of each pillar, respectively. After performing the simulation, the admittance curves for the three positions are obtained, as shown in Figure 4.

Comparing the two graphs, it is evident that the conductance curves of elements in the 1-1-3-type piezoelectric array are largely unaffected by their positions, with the resonance frequency stable at 180 kHz and conductance stable at 0.325 mS, indicating high consistency. In contrast, the conductance curves of elements in the 1-3-type piezoelectric array are significantly affected by their positions, with resonance frequencies fluctuating between 180 kHz and 200 kHz. Moreover, the peak admittance values of the 1-3-type array are approximately 30% lower than those of the 1-1-3-type array. This is attributed to the fact that the flexible polymer surrounding the piezoelectric mini-columns in the 1-1-3-type composite material reduces the internal clamping effect on the material, decreasing the constraint on the longitudinal vibrations of the piezoelectric columns and thus allowing them to vibrate more freely.

**Figure 5:**
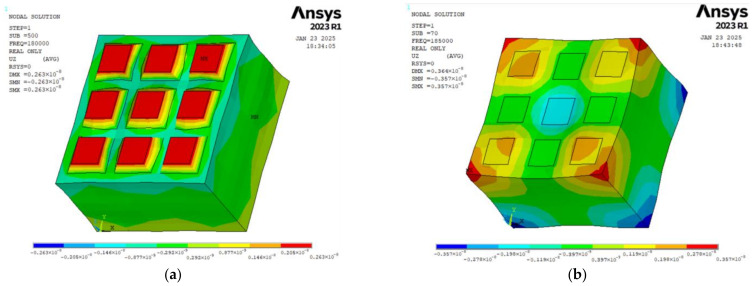
Simulation results of vibration modes. (**a**) Simulation result of the vibration mode for 1-1-3-type piezoelectric composite material. (**b**) Simulation result of the vibration mode for 1-3-type piezoelectric composite material.

**Figure 6:**
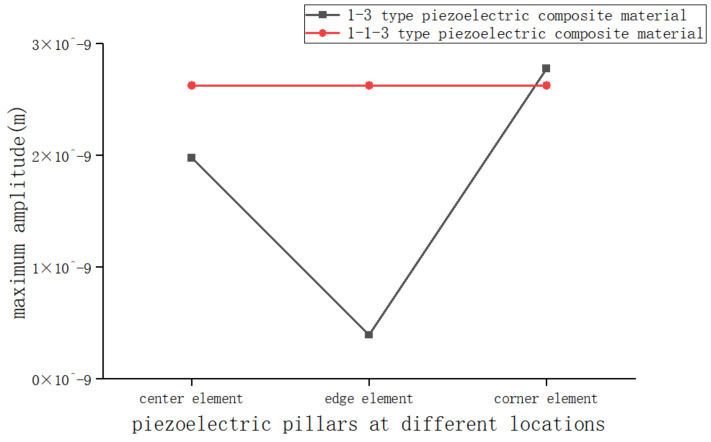
Maximum amplitude at different piezoelectric pillars.

To further analyze the vibration of piezoelectric columns, the vibration modes of the 1-1-3-type and 1-3-type piezoelectric composite materials are obtained, as shown in Figure 5. It can be seen from the figure that the vibration modes of the elements in the 1-1-3-type piezoelectric composite material are more uniform compared to those in the 1-3-type piezoelectric composite material, unaffected by position, and the piezoelectric columns are at the locations of maximum amplitude. According to the selection of piezoelectric pillars in Figure 3, the maximum amplitude curves of piezoelectric pillars at different positions as shown in Figure 6 are drawn. It can be seen that the maximum amplitude at the piezoelectric pillars of the 1-1-3-type piezoelectric composite material is very consistent with that of the 1-3-type piezoelectric composite material.

## 4. Physical Fabrication and Testing of Piezoelectric Composite Materials

**Figure 7:**
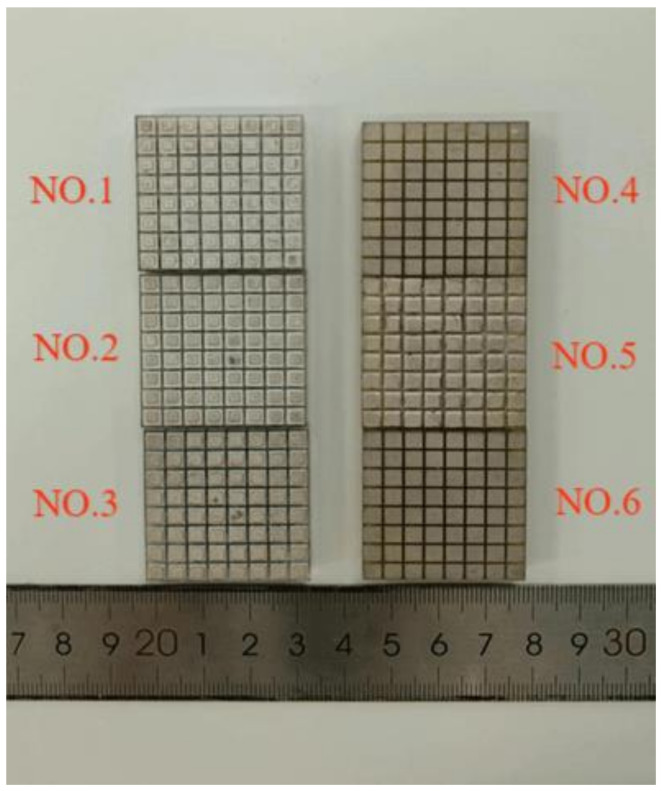
Physical models of 1-3- and 1-1-3-type piezoelectric arrays.

**Figure 8:**
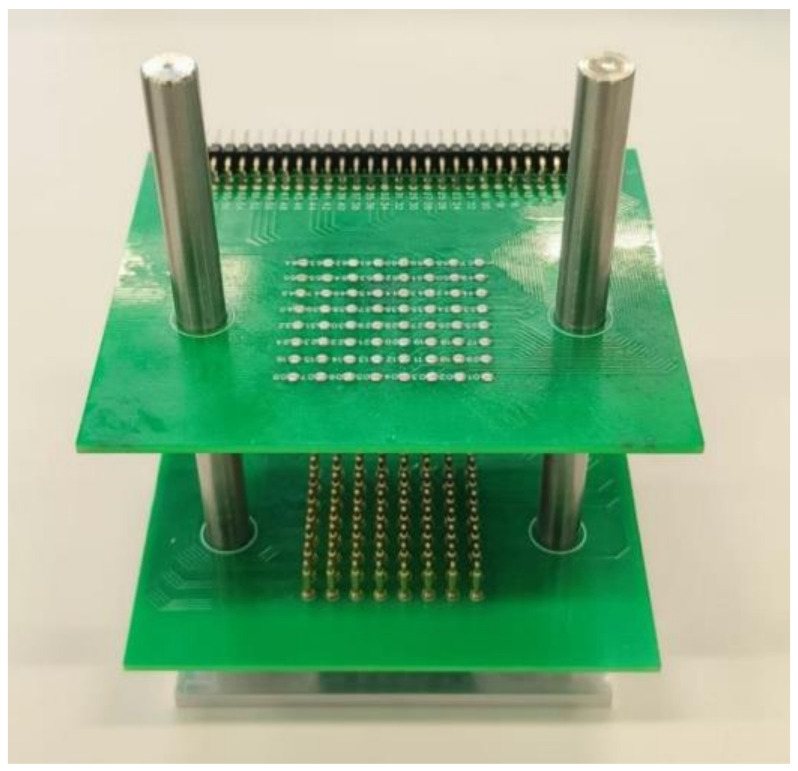
Physical image of the testing fixture.

Piezoelectric composite material arrays were fabricated using PZT-5A-type piezoelectric ceramics from Kunshan Risun Electronic Co., Ltd., Kunshan, China, type 704 silicone rubber from Nanjing University, and epoxy resin. To evaluate the performance of each small column, segmented electrode processing was necessary. The physically fabricated 1-3- and 1-1-3-type piezoelectric arrays are shown in Figure 7, and data testing was performed on these arrays. Traditional testing methods, which mainly involve manual procedures, often struggle to apply uniform stress to each column, resulting in lower testing precision. Therefore, this paper introduces a testing fixture designed to enhance testing accuracy, depicted in Figure 8. This fixture primarily consists of two circuit boards, each featuring 64 spring-loaded pogo pins, and a metal frame. During testing, the piezoelectric composite material is placed between the two circuit boards, with each piezoelectric column making contact with the spring-loaded pogo pins above and below. When the circuit boards are clamped tightly using a locking clamp, the pogo pins compress by an equal distance, exerting uniform stress on each piezoelectric column, thereby achieving higher testing precision.

**Figure 9:**
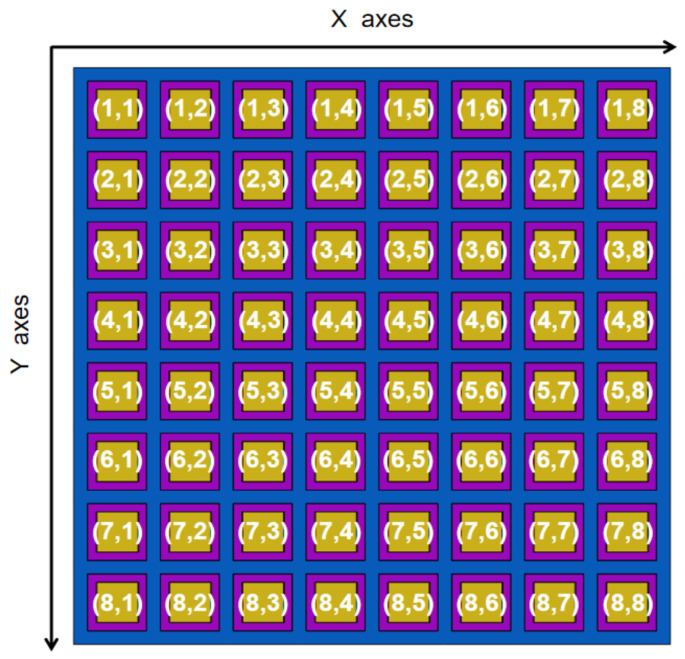
Numbering diagram of each element.

To better display the testing data results, this study assigns numbers to the 8 × 8 array elements based on their coordinates on the X and Y axes. The numbering results are shown in Figure 9.

**Figure 10:**
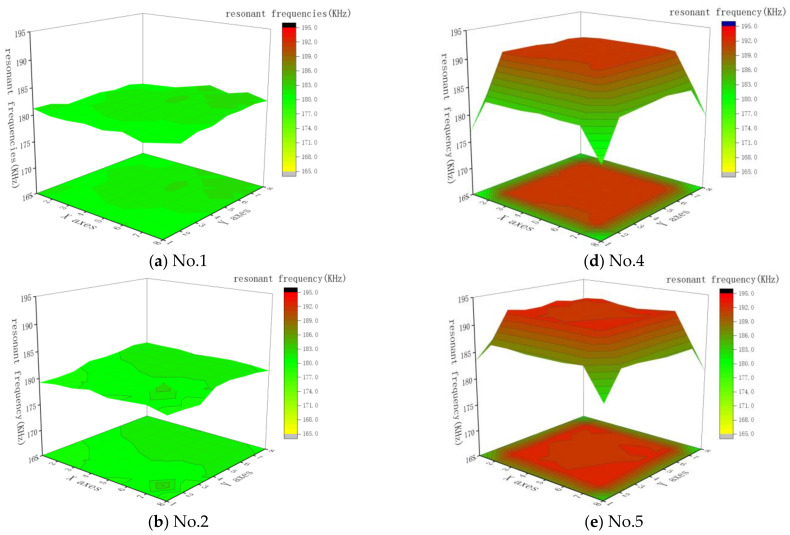

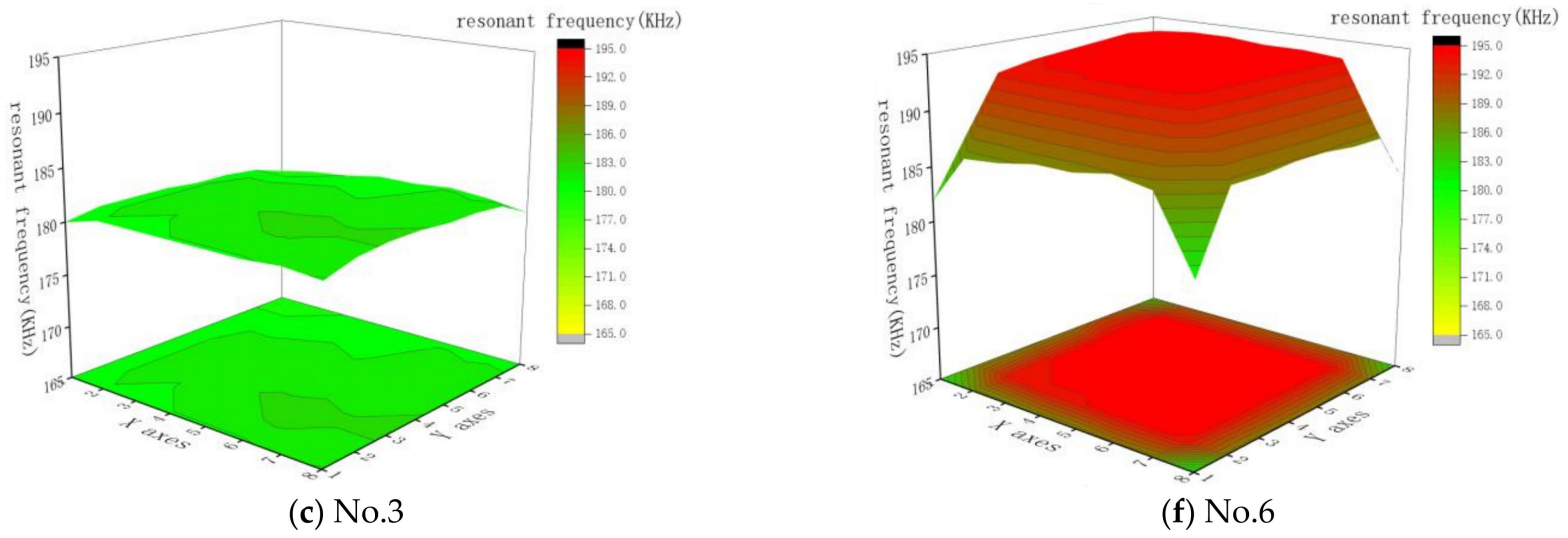
Distribution of resonant frequencies of elements in 1-1-3-type and 1-3-type piezoelectric arrays by position.

The resonant frequency of piezoelectric composite materials refers to the frequency at which they produce the maximum amplitude when operating at their natural frequency. For transducer arrays, maintaining a consistent resonant frequency across the array during acoustic imaging processes allows all elements to operate at the same frequency, reducing the impact of phase differences and enhancing signal quality for clearer imaging results. In this study, an Agilent 4294A impedance analyzer was used to test the resonant frequencies of each element in the fabricated 1-1-3- and 1-3-type piezoelectric array samples. The distribution of resonant frequencies for each element within the arrays is plotted, as shown in Figure 10. Additionally, the mean and standard deviation of the resonant frequencies for each array were calculated, with the results presented in Table 3.

Comparing the resonant frequency data of the 1-1-3-type piezoelectric arrays with the 1-3-type piezoelectric arrays, it is observed that the mean resonant frequency of the 1-1-3-type arrays is consistently lower than that of the 1-3-type arrays when the cross-sectional dimensions of the individual piezoelectric pillars are the same. The average resonant frequency of the three 1-1-3-type arrays produced is around 181 kHz, with a very small standard deviation in resonant frequency, less than 1 kHz. The range is less than 3 kHz, with frequency fluctuations within 1%, indicating a high consistency in resonant frequency. In contrast, for the three 1-3-type piezoelectric arrays produced, the resonant frequencies of the elements on the outer periphery are significantly lower than those of the central elements, with standard deviations in resonant frequency all exceeding 3 kHz. The ranges for numbers 4, 5, and 6 are, respectively, 15.3 kHz, 12 kHz, and 13.8 kHz, with frequency fluctuations within 4%, indicating lower consistency in resonant frequency compared to the 1-1-3-type transducer arrays.

**Figure 11:**
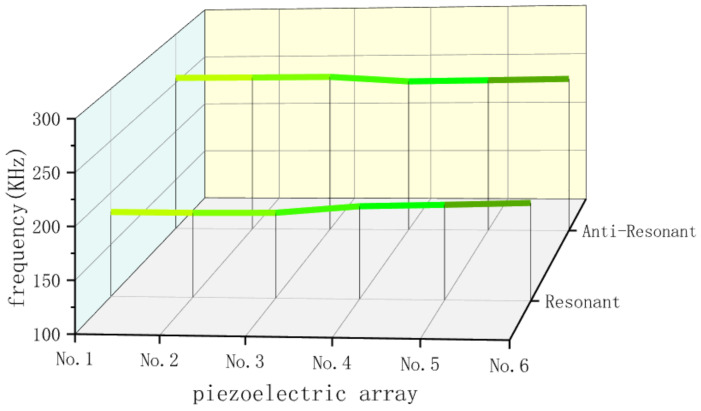
Average resonant frequency and anti-resonant frequency of each piezoelectric array.

The electromechanical coupling coefficient of the elements within the piezoelectric arrays is as follows:

(1) $$k_{eff}=\sqrt{\frac{f_{c}^{2}-f_{s}^{2}}{f_{c}^{2}}}$$

where $f_{s}$ represents the resonant frequency and $f_{c}$ represents the anti-resonant frequency. Using an impedance analyzer, the average anti-resonant frequencies of the piezoelectric composite materials are measured together with the previously measured average resonant frequency data, shown in Figure 11. At the same time, these data are substituted into Equation (1) to calculate the average electromechanical coupling coefficient for each piezoelectric array. The results are shown in Table 4 below. It can be observed that the average electromechanical coupling coefficient of the 1-1-3-type piezoelectric array is generally higher than that of the 1-3-type piezoelectric array.

**Figure 12:**
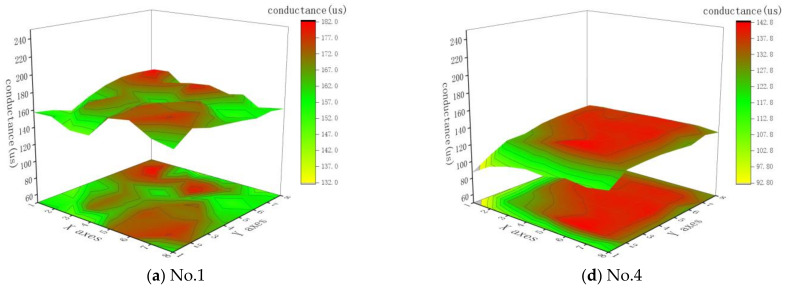

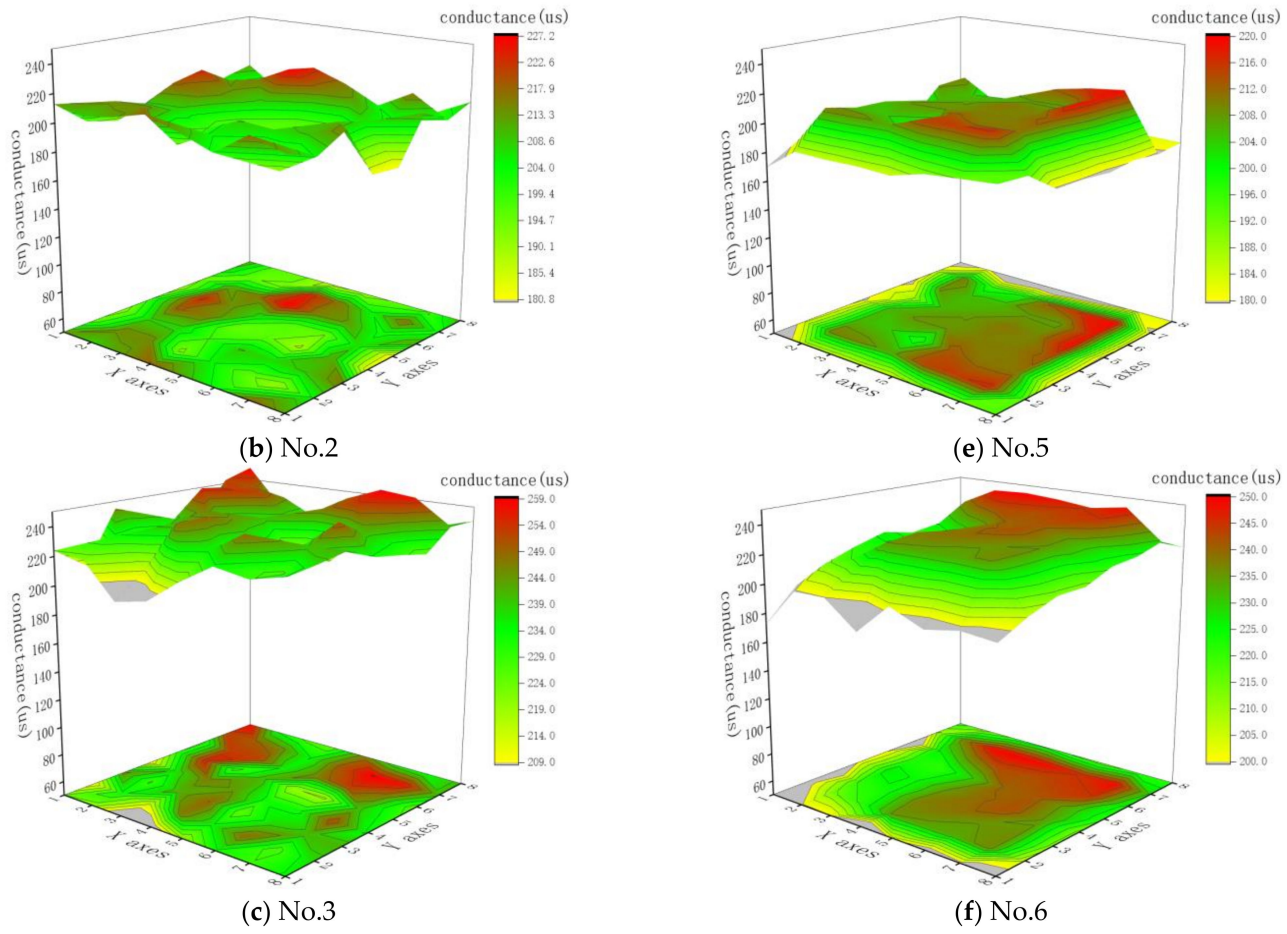
Distribution of conductances of elements in 1-1-3-type and 1-3-type piezoelectric arrays by position.

The conductance value at the resonant frequency of piezoelectric composite materials can also be used to assess the consistency of piezoelectric arrays. In this study, an impedance analyzer was used to measure the G-B curves of the fabricated 1-1-3- and 1-3-type piezoelectric array samples to obtain the peak conductance values at resonance for each element. The distribution of conductance values for each element within the arrays is plotted, as shown in Figure 12. Additionally, the mean and standard deviation of the conductance for each array were calculated, with the results presented in Table 5.

Comparing the conductance data between the 1-1-3- and 1-3-type piezoelectric arrays, it is observed that the average conductance of the 1-1-3-type arrays is consistently higher than that of the 1-3-type arrays with the same dimensions of piezoelectric pillars, which aligns with the simulation results. The standard deviation of conductance in the 1-1-3-type arrays is also lower than that in the 1-3-type arrays. The ranges of conductance for numbers 1, 2, and 3 are, respectively, 37.7 µS, 49.1 µS, and 61.2 µS, with fluctuations within ±11.6%, ±12.0%, and ±13.4%, respectively. For numbers 4, 5, and 6, the conductance ranges are, respectively, 55.4 µS, 58.8 µS, and 77.3 µS, with fluctuations within ±24.2%, ±15.0%, and ±18.3%, respectively. Clearly, when the piezoelectric pillar dimensions are the same, the 1-1-3-type piezoelectric arrays demonstrate better conductance consistency than the 1-3-type arrays.

Observations from Figure 7 and Figure 8, which showcase the distribution of resonant frequency and conductance data for the 1-3-type piezoelectric arrays, reveal a pattern where the data are “higher in the middle and lower on the outer ring”. The performance parameters change significantly with position.

## 5. Conclusions

This paper designed various piezoelectric composite material structures and conducted finite element simulations for 1-1-3- and 1-3-type piezoelectric composites using ANSYS software (2323 R2). By analyzing the changes in the conductance curves and vibration modes with respect to position, it was concluded that the 1-1-3-type piezoelectric composite material exhibits better consistency among its elements. Furthermore, physical fabrication and testing demonstrated that the resonance frequency parameters of elements in the three versions of the 1-1-3-type piezoelectric composites fluctuated within ±1%, which is a 3% reduction in fluctuation compared to the 1-3-type piezoelectric composites, indicating better resonance frequency consistency. Additionally, the conductance parameters of elements in the 1-1-3-type piezoelectric composites also showed reduced fluctuations of 12.6%, 3%, and 4.9% compared to those in the 1-3-type piezoelectric composites, confirming better conductance consistency and corroborating the simulation results. Moreover, the electromechanical coupling coefficient of the 1-1-3-type piezoelectric composites also showed an approximate increase of 7.5% compared to the 1-3-type piezoelectric composites. Therefore, the 1-1-3-type piezoelectric composite material, with its superior consistency, holds promising prospects for application in high-frequency imaging sonar systems.

## Figures and Tables

**Table 1:** Material properties of piezoelectric phase.

Parameters	PZT-5A
$\rho (kg/m^{3})$	7750
$c_{11}^{E}(10^{-10}N/m^{2})$	12.1
$c_{12}^{E}(10^{-10}N/m^{2})$	7.54
$c_{13}^{E}(10^{-10}N/m^{2})$	7.52
$c_{33}^{E}(10^{-10}N/m^{2})$	11.1
$c_{44}^{E}(10^{-10}N/m^{2})$	2.11
$S_{11}^{E}(10^{-12}m^{2}/N)$	16.4
$S_{12}^{E}(10^{-12}m^{2}/N)$	−5.74
$S_{13}^{E}(10^{-12}m^{2}/N)$	−7.22
$S_{33}^{E}(10^{-12}m^{2}/N)$	18.8
$d_{31}$(10^−12^C/N)	470
$d_{33}$(10^−12^C/N)	−171
$e_{15}$(C/m^2^)	12.3
$e_{31}$(C/m^2^)	−5.4
$e_{33}$(C/m^2^)	15.8
$\varepsilon_{11}$($10^{-11}F/m$)	811.026
$\varepsilon_{33}$($10^{-11}F/m$)	734.88

**Table 2:** Table of polymer phase material properties.

	Parameters	Poisson’s Ratio	Young’s Modulus/(Pa)	$\mathrm{Density}/(\mathrm{kg}\cdot m^{-3}$)
Material				
Silicone Rubber		0.48	2.55 ×$\;10^{6}$	1000
Epoxy Resin		0.35	3.60 ×$\;10^{9}$	1050

**Table 3:** Statistical data of resonant frequencies for 8 × 8 elements in 1-1-3-type and 1-3-type piezoelectric arrays by serial number.

	Number	1-1-3-Type Piezoelectric Array			1-3-Type Piezoelectric Array		
Resonant Frequency		No.1	No.2	No.3	No.4	No.5	No.6
Average (KHz)		181.257	180.739	181.125	187.614	189.250	191.252
Standard Deviation (KHz)		0.627	0.733	0.693	4.298	3.382	3.794

**Table 4:** Average Electromechanical Coupling Coefficients and Related Parameters for 1-1-3- and 1-3-Type Piezoelectric Arrays.

	Number	1-1-3-Type Piezoelectric Array			1-3-Type Piezoelectric Array		
Average Data		No.1	No.2	No.3	No.4	No.5	No.6
Resonant Frequency (KHz)		181.257	180.739	181.125	187.614	189.250	191.252
Anti-Resonant Frequency (KHz)		255.151	255.036	255.144	250.061	250.728	251.566
Electromechanical Coupling Coefficient		0.704	0.706	0.704	0.661	0.656	0.649

**Table 5:** Statistical data of conductances for 8×8 elements in 1-1-3-type and 1-3-type piezoelectric arrays.

	Number	1-1-3-Type Piezoelectric Array			1-3-Type Piezoelectric Array		
Conductance		No.1	No.2	No.3	No.4	No.5	No.6
Average(us)		165.140	205.058	236.017	128.060	196.617	222.780
Standard Deviation(us)		8.884	10.611	13.372	13.342	15.566	18.458

## Data Availability

The raw data supporting the conclusions of this article will be made available by the authors on request.
